# Highly Pathogenic Influenza A(H5Nx) Viruses with Altered H5 Receptor-Binding Specificity

**DOI:** 10.3201/eid2302.161072

**Published:** 2017-02

**Authors:** Hongbo Guo, Erik de Vries, Ryan McBride, Jojanneke Dekkers, Wenjie Peng, Kim M. Bouwman, Corwin Nycholat, M. Helene Verheije, James C. Paulson, Frank J.M. van Kuppeveld, Cornelis A.M. de Haan

**Affiliations:** Utrecht University, Utrecht, the Netherlands (H. Guo, E. de Vries, J. Dekkers, K.M. Bouwman, M.H. Verheije, F.J.M. van Kuppeveld, C.A.M. de Haan);; The Scripps Research Institute, La Jolla, California, USA (R. McBride, W. Peng, C. Nycholat, J.C. Paulson)

**Keywords:** influenza A virus, viruses, H5Nx virus, virus clade 2.3.4.4, influenza, highly pathogenic, receptor binding, emergence, hemagglutinin, phylogeny, H5 protein, fucosylated sialosides

## Abstract

Emergence and intercontinental spread of highly pathogenic avian influenza A(H5Nx) virus clade 2.3.4.4 is unprecedented. H5N8 and H5N2 viruses have caused major economic losses in the poultry industry in Europe and North America, and lethal human infections with H5N6 virus have occurred in Asia. Knowledge of the evolution of receptor-binding specificity of these viruses, which might affect host range, is urgently needed. We report that emergence of these viruses is accompanied by a change in receptor-binding specificity. In contrast to ancestral clade 2.3.4 H5 proteins, novel clade 2.3.4.4 H5 proteins bind to fucosylated sialosides because of substitutions K222Q and S227R, which are unique for highly pathogenic influenza virus H5 proteins. North American clade 2.3.4.4 virus isolates have retained only the K222Q substitution but still bind fucosylated sialosides. Altered receptor-binding specificity of virus clade 2.3.4.4 H5 proteins might have contributed to emergence and spread of H5Nx viruses.

Highly pathogenic avian influenza A(H5N1) viruses have caused major economic losses in the poultry industry and might cause zoonotic infections. Recently, a novel H5 virus (clade 2.3.4.4) has emerged (*1*–*4*) and shown unprecedented intercontinental spread. Whereas highly pathogenic avian influenza A(H5N1) viruses previously detected in poultry in the Americas are believed to have descended from low pathogenicity viruses in this region (*5*), clade 2.3.4.4 viruses were the first highly pathogenic avian influenza virus of the A/goose/Guangdong/1/1996 lineage to appear in North America (*2*–*4*) during the winter of 2014–2015. Emergence of these novel viruses resulted in culling of >7 million turkeys and 42 million chickens in the United States (*6*). In Asia, 13 (mostly lethal) cases of human infection with clade 2.3.4.4 H5N6 viruses have been reported (*7*). Emergence of new viruses with clade 2.3.4.4 hemagglutinin (HA) that infect poultry and humans emphasizes the need for detailed characterization of molecular properties of these viruses.

Influenza A viruses are subtyped according to their envelope glycoproteins HA and neuraminidase (NA): 16 HA and 9 NA subtypes are found in aquatic birds and constitute the animal influenza A virus reservoir. Viruses with H5 or H7 subtypes occasionally acquire a multibasic cleavage site in their HA (*8*), which results in a highly pathogenic phenotype. In general, highly pathogenic H5 viruses have the N1 subtype (H5N1). In contrast, the novel highly pathogenic H5 clade 2.3.4.4 viruses have reassorted with different NA subtypes, including N1, N2, N3, N5, N6, and N8 (*1*,*9**–**17*).

HA proteins bind to sialoside receptors on the host cell surface. Avian and human influenza A viruses prefer binding to sialic acids linked to a penultimate galactose by an α2-3 or α2-6 linkage, respectively (*18*). Type and number of internal monosaccharides and their linkages determine fine specificity of virus receptors (*19**,**20*). NA removes sialic acids from glycans, which enables virus particles to be released from the cell surface after assembly and from decoy receptors (e.g., in mucus). The balance between activities of HA and NA proteins has a critical role in optimal viral fitness, tropism, and transmission (*21*).

Changes in HA receptor-binding properties might affect virus host range and within-host virus properties. These changes might have contributed to the remarkable spread of clade 2.3.4.4 viruses. Although a recent study (*22*) reported enhanced avidity of H5N6 viruses for human-type receptors, recombinant clade 2.3.4.4 highly pathogenic influenza A virus H5 proteins from virus isolates in North America show a strict avian receptor-binding preference (*23*). We compared receptor-binding properties for clade 2.3.4.4 H5 proteins from an H5N8 virus from Europe with those for an early ancestral clade 2.3.4 H5 protein from an H5N1 virus to identify differences in these properties.

## Materials and Methods

### Genes, Expression Vectors, and Protein Expression and Purification

We cloned codon-optimized H5-encoding cDNAs (GenScript, Piscataway, NJ, USA) of H5N1 A/wild duck/Hunan/211/2005 (GenBank accession no. EU329186.1) and H5N8 A/chicken/Netherlands/14015526/2014 (GISAID, https://platform.gisaid.org**, accession** no. EPI_ISL_167905) in pCD5 expression vectors flanked by signal peptide-, GCN4- isoleucine-zipper trimerizaton motif-, and Strep-tag II-encoding sequences. Clones were mutagenized when indicated, expressed, and purified as described (*24*).

### HA Receptor-Binding Assays

We assessed binding of HA to fetuin and transferrin (Sigma, St. Louis, MO, USA) and glycan arrays as described (*24*). Biolayer interferometry was performed by using Octet QK (ForteBio, Menlo Park, CA, USA) and in-house–synthesized saccharides NeuAcα2-3Galβ1-4GlcNAcβ1-3Galβ1-4GlcNAc (3′SLNLN), NeuAcα2-6Galβ1-4GlcNAcβ1-3Galβ1-4GlcNAc (6′SLNLN), NeuAcα2-3Galβ1-4(Fucα1-3)GlcNAc (3′SLe^X^), and NeuAcα2-6Galβ1-3GlcNAc (3′SLN) coupled to LC-LC-biotin. Streptavidin sensors were loaded with 0.1 μmol/L of glycan for 15 min. We assessed association of HA proteins (0.2 μg/μL) with StrepMAB-Classic (IBA GmbH, Göttingen, Germany) at a molar ratio of 2:1 and performed binding of HA to tissues as described (*25*), except that H5 proteins were precomplexed with StrepMAB-Classic conjugated to horseradish peroxidase (IBA GmbH) and goat antimouse IgG (heavy plus light chain) conjugated to horseradish peroxidase (Invitrogen, Carlsbad, CA, USA). We performed immunohistochemical analysis with 3′SLe^X^-specific antibody KM93 (*26*) (EMD Millipore, Darmstadt, Germany) at a 1:500 dilution by using standard procedures, including antigen retrieval (*27*) on an avian intestinal tissue microarray (*28*).

### Modeling

The crystal structure of H5 protein from A/Vietnam/1194/2004 (H5N1) virus was complexed with 3′SLe^X^ (Protein Data Bank accession no. 3ZNM 28) (*29*) and used as a template for modeling the structures of A/wild duck/Hunan/211/2005 and A/chicken/Netherlands/14015526/2014 viruses with SWISS-MODEL (*30*). Subsequent energy minimizations were not necessary because inspection of modeled structures by using GROMOS (http://www.gromacs.org/Documentation/Terminology/Force_Fields/GROMOS) showed no unfavorable energy interactions. Superpositioning of Cα-backbone atoms of residues lining the receptor-binding site of A/wild duck/Hunan/211/2005 (H5N1) or A/chicken/Netherlands/14015526/2014 (H5N8) viruses with A/Vietnam/1194/2004 (H5N1) virus showed that the root mean square deviation of superpositioned atoms was <0.2 Å for both viruses. Molecular interactions were further examined by using Swiss-Pdb Viewer (*31*).

### Receptor-Binding Properties of Different H5 Proteins 

**Figure 1:**
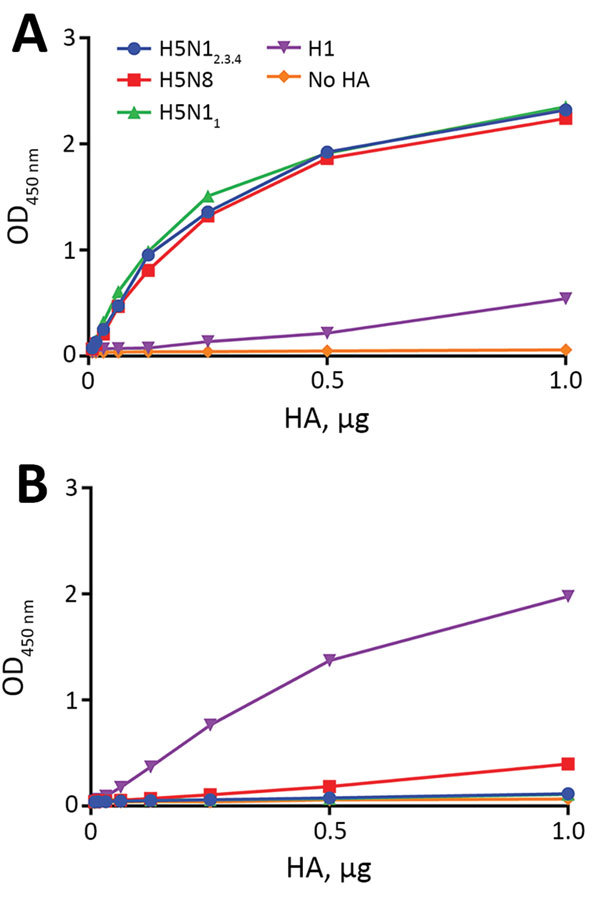
Binding of influenza A virus hemagglutinins to A) fetuin and B) transferrin. Limiting dilutions of soluble H5 trimers complexed with horseradish peroxidase−conjugated antibodies were used in a fetuin- or transferrin-binding assay. Optical density at 450 nm (OD_450_) corresponds to binding of HA to glycoproteins. HA, hemagglutinin; H5N1_2.3.4_, novel H5N1 virus clade 2.3.4; H5N1_1_, H5N1 virus clade 1.

We compared receptor-binding properties of an H5 protein derived from an early clade 2.3.4 H5N1 virus isolate (A/wild duck/Hunan/211/2005) with a clade 2.3.4.4 isolate (H5N8 A/chicken/Netherlands/14015526/2014) detected in Europe (referred to as H5N1_2.3.4_ and H5N8 HA proteins, respectively) by using recombinant soluble HA proteins (*24*). H5 proteins were analyzed for their binding to fetuin and transferrin (Figure 1). Fetuin contains α2-3–linked and α2-6–linked sialiosides at a ratio of 2:1 (*32**,**33*), but transferrin contains only α2-6–linked sialosides (*34*). We used a clade 1 H5 protein (H5N1 A/Viet Nam/1203/2004; referred to as H5N1_1_) and an H1 protein of a human seasonal H1N1 virus (A/Kentucky/UR06–0258/2007; referred to as H1) with known receptor-binding properties as controls (*24**,**27*).

## Results

### Receptor-Binding Properties of Different H5 Proteins 

All H5 proteins efficiently bound fetuin. However, only H5N8 virus HA showed limited binding to transferrin, which indicated that H5N1_2.3.4_ and H5N8 HA proteins prefer binding to α2-3–linked sialic acids similarly as H5N1_1_ HA (*27*). The H1 protein bound fetuin to a lower extent than the H5 proteins and bound transferrin, which was consistent with this protein preferentially binding α2-6–linked sialosides (*24*).

**Figure 2:**
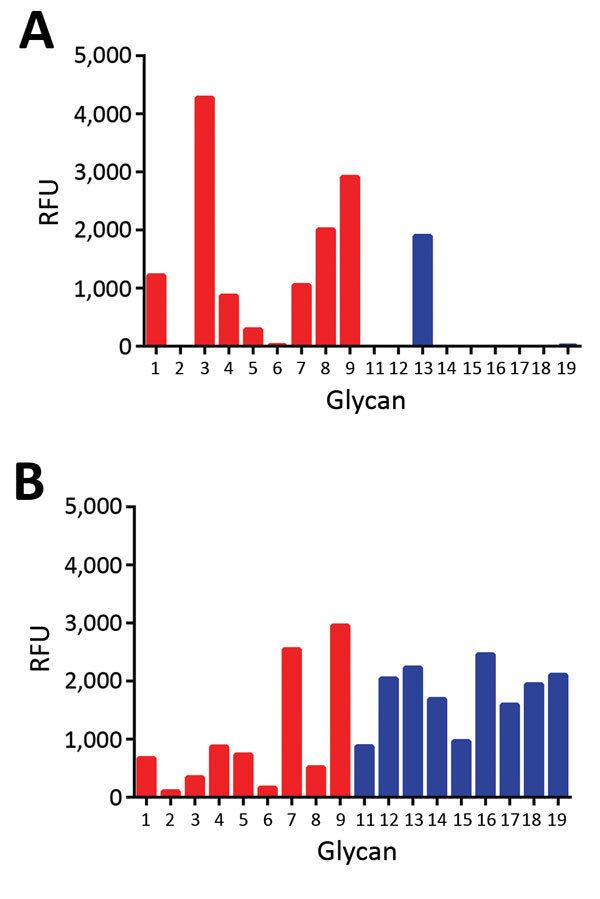
Glycan array analysis of recombinant H5 proteins of influenza A viruses. A) Wild-type H5N1_2.3.4_ (KS) and B) H5N8 (QR) H5 proteins were applied to the glycan array precomplexed with StrepMAB-classic (IBA GmbH, Göttingen, Germany) and fluorescent secondary antibodies. Letters in parentheses indicate amino acids at positions 222 and 227. Binding of hemagglutinins is indicated in relative fluorescence units (RFU). Binding is shown to sialylated glycans present in the array for nonfucosylated (glycans 1–9; red bars) and fucosylated (glycans 11–19; blue bars) forms. Glycan numbers indicated on the *x*-axes correspond to glycan structures shown in Figure 3. H5N1_2.3.4_, novel H5N1 virus clade 2.3.4.

Receptor fine specificity of H5N1_2.3.4_ and H5N8 HAs was determined by using glycan array analysis. The H5 proteins bound to a range of mono- and bi-antennary α2-3−linked glycan structures corresponding to N- and O-linked sialosides (Figure 2). Both proteins did not bind to α2-6−linked sialosides. The specificity of the H5N1_2.3.4_ HA was similar to that of its H5N1_1_ ancestor (*27*) (Technical Appendix Figure). However, glycan array analysis showed a fucosylation-specific change in receptor fine specificity for H5N8 HA.

**Figure 3:**
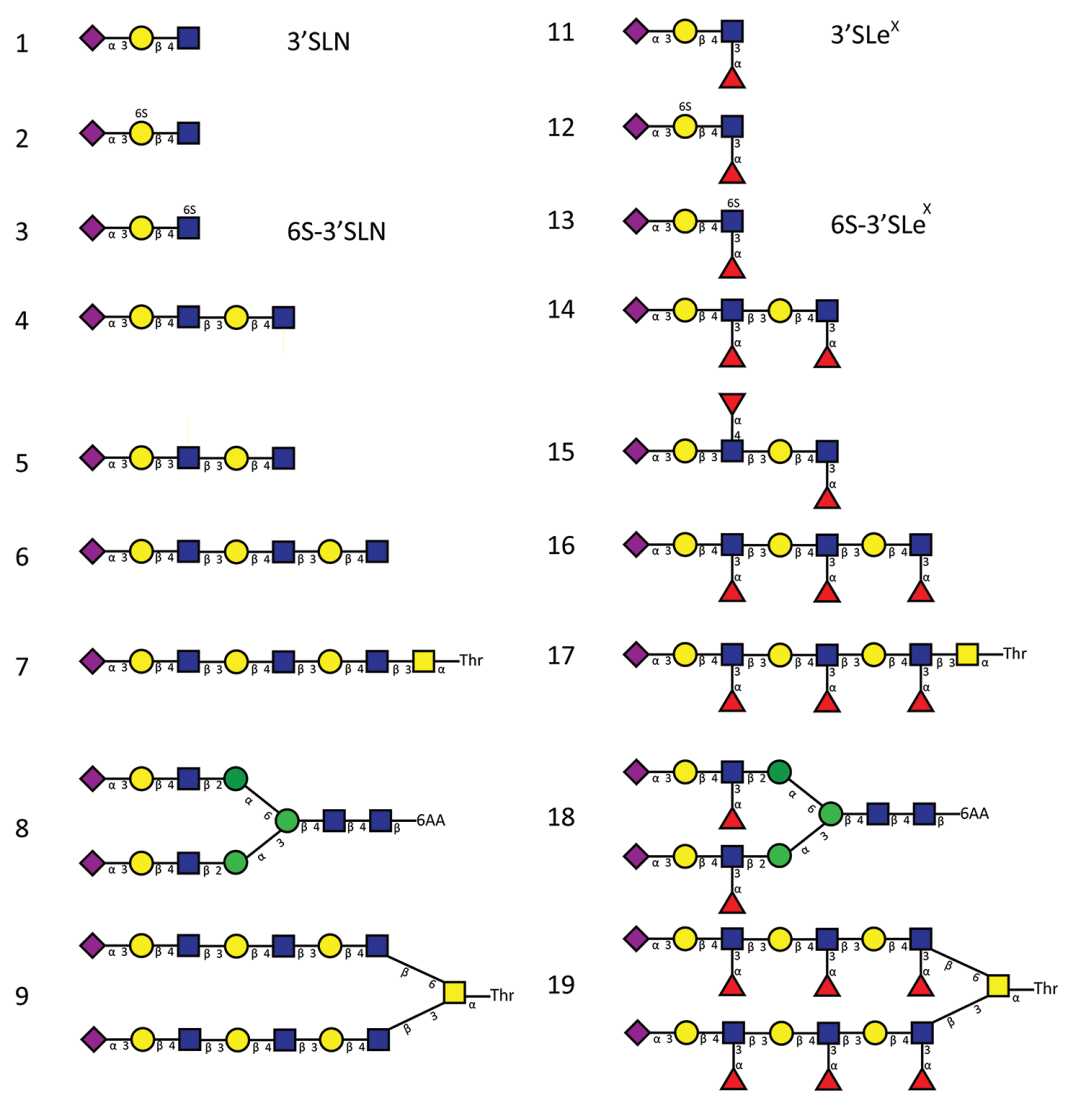
Glycan structures of influenza A viruses. Structures are shown for sialylated glycans present in the array in nonfucosylated (glycans 1–9) and fucosylated (glycans 11–19) forms and binding by hemagglutinins is shown in Figures 2 and 7. Glycans 1 and 11 correspond to 3′SLN (nonfucosylated glycan) and 3′SLe^X^ (fucosylated form of 3′SLN), respectively. Similarly, glycans 3 and 13 correspond to 6-O-sulfo 3′SLN (6S-3′SLN) and 6-O-sulfo 3′SLe^X^ (6S-3′SLe^X^), respectively. Blue squares, N-acetylglucosamine; yellow circles, galactose; green circles, mannose; purple diamonds, sialic acid; red triangles, fucose. H5N1_2.3.4_, novel H5N1 virus clade 2.3.4.

**Figure 4:**
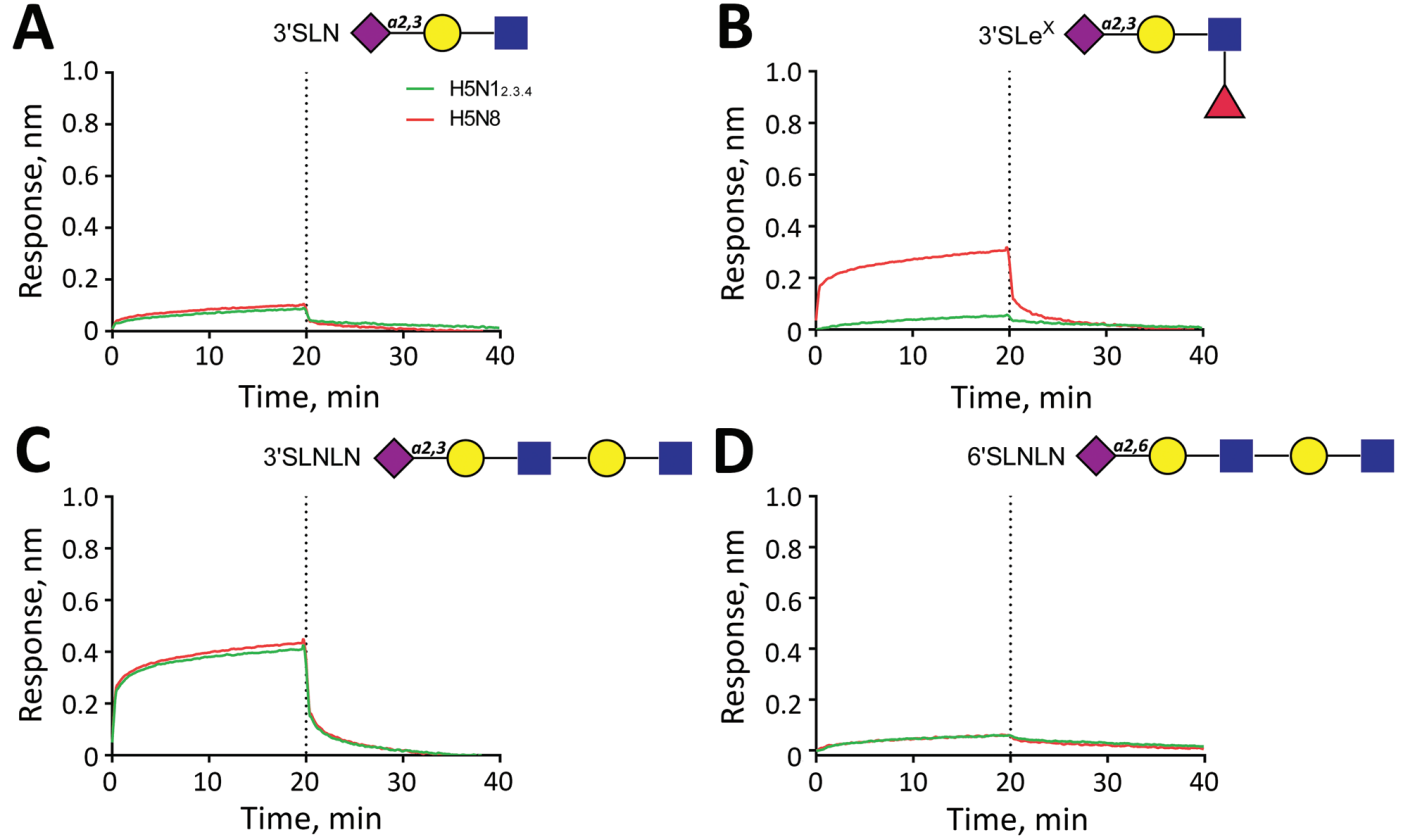
Analysis of binding of influenza A virus H5N1_2.3.4_ and H5N8 hemagglutinins to sialylated glycans by using biolayer interferometry. A) 3′SLN, B) 3′SLe^X^, C) 3′SLNLN, D) 6′SLNLN. After complexing biotinylated glycans with streptavidin sensors, association and subsequent dissociation of H5 proteins complexed with StrepMAB-classic (IBA GmbH, Göttingen, Germany) was determined. Blue squares, N-acetylglucosamine; yellow circles, galactose; purple diamonds, sialic acid; red triangles, fucose. The dotted lines at the 20-min time points distinguish the association and dissociation phases. H5N1_2.3.4_, novel H5N1 virus clade 2.3.4.

We compared binding to 2 sets of α2-3−linked sialosides that differed only in the absence (glycans 1–9) or presence (glycans 11–19) of fucose at the GlcNAc of lactosamine repeats (Figures 2, 3). H5N1_2.3.4_ HA bound only to 1 fucosylated glycan (glycan no. 13; 6-sulfo 3′Sialyl Lewis X [6S-3′SLe^X^]). In contrast, H5N8 HA bound to all α1-3−fucosylated glycan, as well as the single α1-4−fucosylated glycan (glycan no. 15). We also analyzed binding of these proteins to different glycans by using biolayer interferometry (Figure 4). Although H5N1_2.3.4_ and the H5N8 HAs showed similar binding properties to 3′SLNLN, 6’SLNLN, and 3′SLN, they clearly differed in their binding to 3′SLe^X^, which is consistent with results of glycan array analysis (Figure 2).

### Phylogenetic Analysis of Clade 2.3.4.4 H5 Proteins

**Figure 5:**
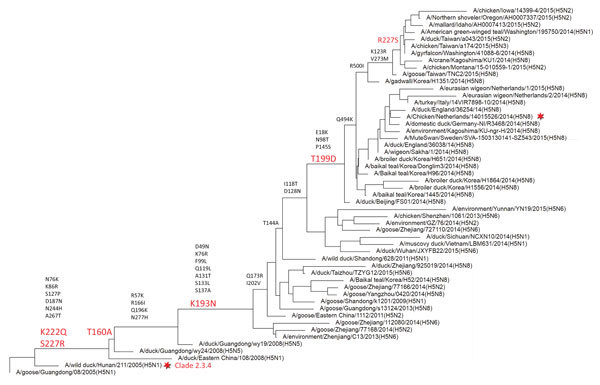
Phylogenetic analysis of influenza A virus clade 2.3.4.4 H5 proteins. A 362-aa full-length hemagglutinin (HA) sequence for H5 clade 2.3.4.4 was obtained from GenBank and the GISAID database **(**http://platform.gisaid.org**).** An HA protein tree was constructed by using the PHYLIP neighbor-joining algorithm (https://ugene.net/wiki/display/UUOUM/PHYLIP+Neighbor-Joining) and the F84 distance matrix. This tree was used to construct a guide tree with 52 HA sequences representing all branches of the tree. These sequences were used to construct a summary tree of similar topology as the guide tree. Items above the branches indicate key residues that differ between different branches. Items in red above the branches indicate mutations introduced in this study. The HA protein tree is rooted by an early clade 2.3.4 isolate (A/goose/Guangdong/08). H5N1_2.3.4_ and H5N8 HA proteins used in this study are indicated by red stars. H5N1_2.3.4_, novel H5N1 virus clade 2.3.4.

To identify residues responsible for these differences in receptor binding, we mapped the amino acid substitutions along the trunk of an HA phylogenetic tree (Figure 5). Clade 2.3.4.4 viruses first emerged in 2008 (*35*). Eight amino acid substitutions, still maintained in the recently emerging clade 2.3.4.4 viruses, characterize the transition from the most closely related clade 2.3.4 ancestor (A/wild duck/Hunan/211/2005) (H5N1). A number of these amino acid substitutions in 2,562 HAs from highly pathogenic influenza A(H5N1) viruses were isolated during 1996−2015 (excluding clade 2.3.4.4 viruses) (Table 1). **S**ubstitutions K222Q and S227R (H3 numbering used here, corresponding to K218Q and S223R in H5 numbering), which have rarely occurred (0.08% and 0.9%, respectively), were found in the receptor-binding site. In the course of further evolution, a glycosylation site at the head domain at position 160 is lost, and there are 2 other unique substitutions (K193N, T193D) in the vicinity of the receptor-binding site. The Ser residue at position 227 present in H5N1_2.3.4_ HA was again found in more recent clade 2.3.4.4 H5-containing viruses that have been detected in North America.

**Table 1:** Amino acids in hemagglutinins from 2,562 highly pathogenic influenza A(H5N1) viruses isolated during 1996−2015 (excluding clade 2.3.4.4 viruses)

Position and amino acid	No. occurrences
18K	119
49N	301
57K	481
76K	16
76R	0
86R	5
98T	32
99L	57
118T	1
119L	0
123R	40
127P	329
128N	276
131T	49
133L	1,029
137A	545
144A	4
145S	1,873
160A	1,467
166I	160
173R	67
187N	117
193N	4
196K	183
199D	0
202V	75
222Q	2
227R	23
244H	316
267T	1,805
273M	10
277H	1
494K	0
500I	0

### Receptor-Binding Properties of Mutant H5 Proteins

**Figure 6:**
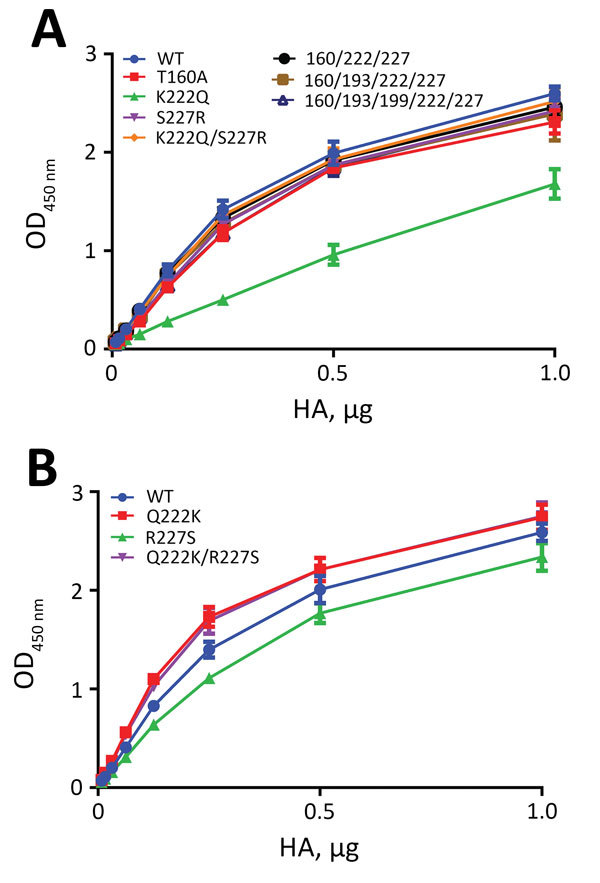
Binding of influenza A virus mutant H5N1_2.3.4_ HA (A) and H5N8 HA (B) to fetuin. Binding was assayed as described in the legend to Figure 1. Mutated residues are indicated. 160/222/227, 160/193/222/227, and 160/193/199/222/227 refer to T160A/K222Q/S227R, T160A/K193N/K222Q/S227R and T160A/K193N/T199D/K222Q/S227R substitutions in H5N1_2.3.4_ HA, respectively. Optical density at 450 nm (OD_450_) corresponds to binding of HA to glycoproteins. WT, wild-type; HA, hemagglutinin. H5N1_2.3.4_, novel H5N1 virus clade 2.3.4.

We introduced the amino acid substitutions in H5N8 HA into H5N1_2.3.4_ HA and analyzed for their effects on receptor binding. Substitutions T160A or S227R did not affect fetuin binding (Figure 6, panel A), but K222Q reduced binding ≈2-fold. Reduction of binding was not observed when substitution K222Q was combined with S227R. Introduction of additional substitutions at positions 160, 193, and 199 in the order in which they occurred during evolution of clade 2.3.4.4 HAs did not affect fetuin binding.

**Figure 7:**
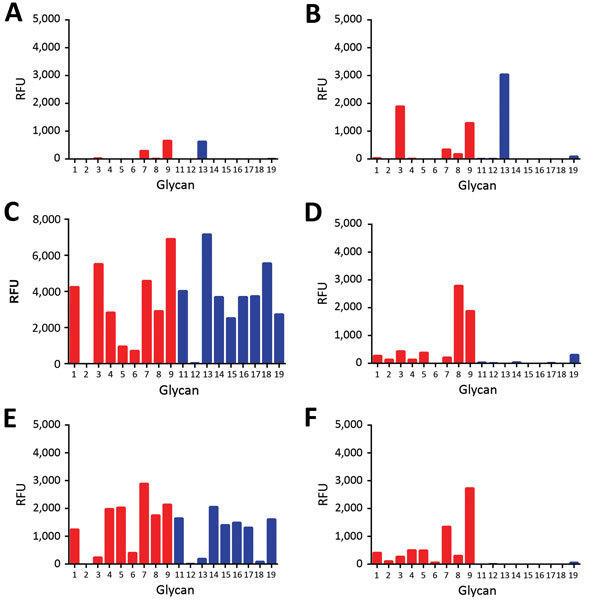
Glycan array analysis of influenza A virus mutant H5 proteins. A) mutant H5N1_2.3.4_ K222Q (QS); B) mutant H5N1_2.3.4_ S227R (KR); C) mutant H5N1_2.3.4_ K222Q/S227R (QR); D) H5N8 Q222K (KR); E) R227S (QS); F) Q227R/R227S (KS). Proteins were applied to the glycan array as detailed in the legend to Figure 2. Letters in parentheses indicate amino acids at positions 222 and 227. Binding of hemagglutinins is indicated in relative fluorescence units (RFU). Binding is shown to sialylated glycans present in the array in nonfucosylated (glycans 1–9; red bars) and fucosylated (glycans 11–19; blue bars) forms. Glycan numbers indicated on the *x*-axes correspond to glycan structures shown in Figure 3. H5N1_2.3.4_, novel H5N1 virus clade 2.3.4.

We studied the receptor fine specificity of these proteins by using glycan array analysis. Substitution T160A did not change receptor fine specificity (Technical Appendix Figure). In comparison with wild-type H5N1_2.3.4_ HA (Figure 2, panel A), substitution K222Q (Figure 7, panel A) strongly decreased binding. However, substitution S227R (Figure 7, panel B) had a more specific negative effect. In contrast, combined substitutions K222Q and S227R (Figure 7, panel C) enhanced binding to the glycans bound by the wild-type H5N1_2.3.4_ HA (Figure 2, panel A) and resulted in additional binding of fucosylated glycans that are also bound by H5N8 HA (Figure 2, panel B). Additional introduction of mutations found at positions 160, 193, and 199 in the background of Q222- and R227-containing H5N1_2.3.4_ HA did not affect receptor fine specificity (Technical Appendix Figure). We conclude that the combination of substitutions K222Q and S227R, already present in the earliest clade 2.3.4.4 H5Nx viruses (Figure 5), is largely responsible for the different receptor-binding properties of the H5N1_2.3.4_ and the H5N8 HAs.

The branch of clade 2.3.4.4 H5 proteins that contains viruses from Taiwan and North America contains reverse substitution R227S (Figure 5). Therefore, we studied how residues at positions 222 and 227 affects receptor binding in the background of H5N8 HA. Substitutions R227S or Q222K hardly affected fetuin binding (Figure 6, panel B). In the glycan array, the R227S substitution did not affect binding of H5N8 HA to fucosylated sialosides (Figure 7, panel E), but the Q222K substitution, alone or in combination with the R227S substitution, inhibited binding of fucosylated receptors (Figure 7, panels D, F). We conclude that the identity of residue 222 plays a crucial role in binding of fucosylated sialosides, regardless of the background of highly pathogenic influenza A virus H5 protein. However, although R227 is required for binding of fucosylated sialosides in clade 2.3.4 H5, this is not the case for clade 2.3.4.4 H5.

### Modeling of Amino Acid Substitutions in H5 Protein Structure

**Figure 8:**
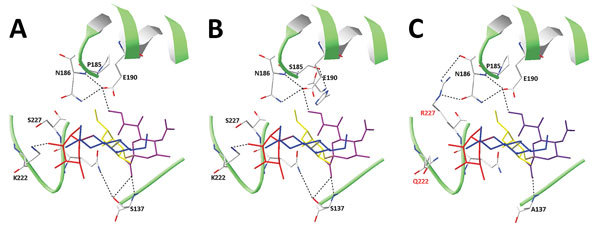
Structural models of influenza A virus H5 proteins complexed with 3′SLe^X^. A) Clade 1 H5 (H5N1_1_ of A/Vietnam/1194/2004) complexed with 3′SLe^X^ (PDB 3ZNM0 (*29*). B) H5N1_2.3.4_ and C) H5N8 hemagglutinins were modeled into the structure shown in panel A as detailed in Materials and Methods. Key amino acids are indicated and shown in a stick representation. C (gray), O (red), and N (blue) in the side chains are colored. SIA, Gal, GlcNAc, and Fuc moieties of 3′SLe^X^ are shown in purple, yellow, blue, and red, respectively. Hydrogen bonds are indicated by dotted lines. H5N1_2.3.4_, novel H5N1 virus clade 2.3.4; H5N1_1_, H5N1 virus clade 1.

We analyzed the interaction of the H5N1_2.3.4_ and the H5N8 HAs with a fucosylated and sialylated tetrasaccharide (3′SLe^X^; glycan no. 3) (Figure 3) by modeling on the structure of a clade 1 highly pathogenic influenza A(H5N1) virus H5 protein (H5N1_1_ A/Vietnam/1194/2004) that was cocrystalized with 3′SLe^X^ (*28*). The 3′SLe^X^ ligand and major parts of the receptor-binding site (190-helix, 130-loop, and 220-loop) is shown in Figure 8. Poor binding of A/Vietnam/1194/2004 HA to 3′SLe^X^ was explained (*29*) by steric hindrance between Lys at position 222 and fucose (Figure 8, panel A). This steric hindrance is maintained in H5N1_2.3.4_ HA but is lost after the K222Q substitution in H5N8 HA (Figure 8, panels B, C).

The effects of aa 227 on binding of fucosylated sialosides might result from the possibility of forming a hydrogen bond between the 220-loop and the amino-terminal end of the 190-helix through the side chains of R227 and N186, thereby influencing the flexibility of the receptor-binding site. At 2 positions that differ between H5N1_2.3.4_ and H5N8 HAs (S137A and S185P), we observed changes in the potential to form hydrogen bonds between major elements of the receptor-binding site (Figure 8). Such changes might affect the interaction of HA with sialic acid-containing glycans and might explain the background-dependent effect of the residue at position 227.

### Binding of H5 Proteins to Avian Tissues

**Figure 9:**
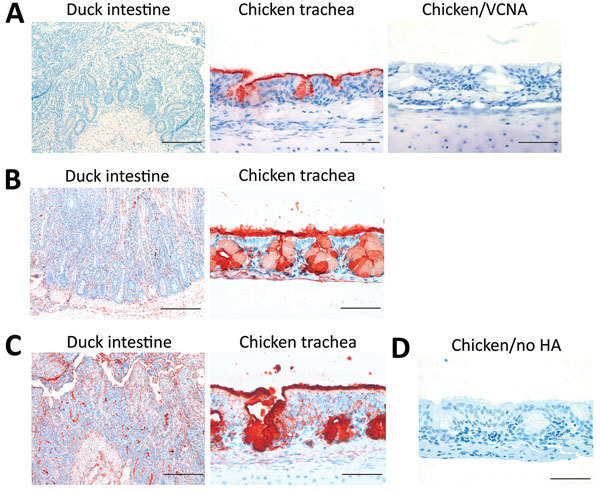
Histochemical analysis of binding of influenza A virus H5 proteins to avian tissues. A) Duck intestine and chicken trachea tissues stained with an antibody specific for 3′SLe^X^ (anti-3′SLe^X^). Tissue sections treated with *Vibrio cholerae* neuraminidase (VCNA) before immunostaining were used as controls. Scale bars indicate 200 μm in left panel and 50 μm in center and right panels. B, C) Duck intestine and chicken trachea tissues incubated with H5 proteins H5N1_2.3.4_ and H5N8 after precomplexing with horseradish peroxidase (HRP)−conjugated antibodies. Scale bars indicate 200 μm in left panel and 50 μm in right panel. D) Chicken trachea tissues incubated with HRP-conjugated antibodies against H5N1_2.3.4_ (no hemagglutinin [HA]) were used as a negative control. H5N1_2.3.4_, novel H5N1 virus clade 2.3.4. Scale bar indicates 50 μm.

We studied binding of H5N1_2.3.4_ and H5N8 H5 proteins to avian tissues that differ in the presence of fucosylated sialosides. An antibody to 3′SLe^X^ bound strongly to epithelial cells of chicken trachea, but not to duck intestinal tissue (Figure 9, panel A), which is consistent with previous findings (*36*). Removal of sialic acids by *Vibrio cholerae* neuraminidase inhibited binding. Tissue derived from another Anseriformes species (Greylag/Canada goose) also did not display 3′SLe^X^, and differential staining results were obtained for intestinal tissues of different Galliformes species (Table 2). The H5 proteins tested efficiently bound chicken trachea (Figure 9, panels B, C) and duck intestines that do not have 3′SLe^X^−containing glycans. Staining of duck tissues with H5N1_2.3.4_ HA was less intense than for H5N8 HA. We conclude that 3′SLe^X^−containing sialosides might affect binding of H5N8 HA to avian tissues but is not essential.

**Table 2:** Detection of 3′SLe^X^ in intestine of avian hosts of influenza A(H5Nx) virus clade 2.3.4.4 subtypes*

Order	Family	Common name	3′SLe^X^ staining of intestine	H5Nx virus infection†
Galliformes	*Phasianidae*	Chicken	+	H5N8/H5N6/H5N2
Galliformes	*Phasianidae*	Turkey	±	H5N8/H5N6
Galliformes	*Phasianidae*	Quail	–	H5N8
Galliformes	*Phasianidae*	Pheasant	+	H5N2
Galliformes	*Phasianidae*	Partridge	–	ND
Galliformes	*Numididae*	Guinea fowl	+	H5N8
Anseriformes	*Anatidae*	Mallard duck	–	H5N8/H5N6/H5N2
Anseriformes	*Anatidae*	Teal	NT	H5N8/H5N6
Anseriformes	*Anatidae*	Swan	NT	H5N8
Anseriformes	*Anatidae*	Greylag/Canada goose	–	H5N8/H5N6
Columbiformes	*Columbidae*	Pigeon	++	H5N6
Falconiformes	*Falconidae*	Gyrfalcon	NT	H5N8
Gruiformes	*Gruidae*	Crane	NT	H5N8
Strigiformes	*Strigidae*	Snowy owl	NT	H5N2
Acipitriformes	*Accipitridae*	Cooper’s hawk/bald eagle	NT	H5N2/H5N8

*ND, not detected; NT, not tested; SLe, sialyl Lewis; –, no visible staining; ±, few cells weakly stained; +, intense staining of cells; ++, intense staining of many cells. †Subtypes identified in different bird species on the basis of data from GenBank and GISAID (http://platform.gisaid.org).

## Discussion

Clade 2.3.4.4 H5Nx viruses have shown unprecedented worldwide spread. Gene reassortments with other influenza A virus genotypes have generated a range of clade 2.3.4.4 viruses containing different NA subtypes, although they harbor an H5 protein that previously was almost exclusively associated with members of a monophyletic clade of N1 proteins descending from early highly pathogenic H5N1 virus isolates. We show that emergence of clade 2.3.4.4 H5Nx viruses is accompanied by a change in HA receptor-binding specificity. Altered receptor-binding properties might affect the balance between HA and NA, enable the virus to acquire different NA subtypes, and might result in altered host range and spreading.

Clade 2.3.4.4 HA in an H5N8 virus from Europe efficiently binds fucosylated sialosides, in contrast to an HA from the ancestral clade 2.3.4 (this study) and older highly pathogenic H5N1 virus HAs (*28**,**37*). We have shown that amino acid substitutions K222Q and S227R in the receptor-binding site of early clade 2.3.4.4 HAs are required for this change in receptor-binding specificity. HA residues K222 and S227 are extremely conserved among all clades of highly pathogenic H5N1 viruses; the double substitution K222Q/S227R was introduced only at the root of clade 2.3.4.4 (Figure 5). Structural analysis of a clade 1 HA indicates that the close proximity of the conserved K222 side chain and the fucose moiety of 3′SLe^X^ most likely destabilizes their interaction (*29*). Modeling indicates that such destabilization is still present in H5N1_2.3.4_ HAs but is absent in H5N8 HAs that contain Q222 and R227 residues (Figure 8).

Introduction of K222Q into H5N1_2.3.4_ HA, which removes the competition with the fucose-moiety (*29*), in itself does not enable binding to fucosylated receptors (Figure 7). However, additional introduction of an arginine at position 227 (double substitution K222Q/S227R) in H5N1_2.3.4_ HA was sufficient to cause a glycan array binding profile nearly identical to that of H5N8 HA. Substitution S227R might result in the 220-loop interacting with the conserved loop at the N terminus of the 190-helix through 2 hydrogen bonds between R227 and N186 (Figure 8, panel C). This interaction, which potentially limits mobility of the 220-loop, might contribute to stabilizing the receptor-binding site in a conformation that enables binding of fucosylated receptors. R227 was required for binding of fucosylated sialosides in the background of H5N1_2.3.4_ HA, but not H5N8 HA (Figure 7). Consistent with our findings, HAs of the Taiwanese/North American branch of clade 2.3.4.4 viruses, which obtained reverse substitution R227S (Figure 5), also efficiently bind fucosylated receptors (*23*).

Analysis of all HA sequences for all avian genotypes available in GenBank showed that the frequency of Q222 and R227, depending on the genotype, is 0 or extremely low. An exception is that Q222 is highly conserved in H7 and H10 genotypes, and R227 is the dominant amino acid in H6 and H13 genotypes. The combination Q222/R227 is present in a 1 clade of low pathogenicity H5N2 viruses represented by A/chicken/Ibaraki/1/05 (H5N2) (*38*), which was shown to be able to bind to fucosylated receptors, and for which residues 222 and 227 were shown to be essential (*39*). The HA of this virus appeared to show reduced binding to nonfucosylated glycans, in contrast to the clade 2.3.4.4 proteins analyzed in this study. However, the contribution of individual residues at these positions for receptor binding was not evaluated.

The isolates of this H5N2 clade in Japan originated from H5N2 viruses in Central America (*38*). Intercontinental transfer of avian influenza viruses is a rare event, drawing a remarkable parallel between 2 viral clades (highly pathogenic H5Nx and low pathogenicity H5N2), both of which have acquired the ability to bind fucosylated receptors and managed to spread intercontinentally. Similar to highly pathogenic H5Nx viruses, low pathogenicity H5N2 viruses might be spread by wild birds, although the possibility could not be ruled out that a virus or vaccine was illegally introduced into Japan from Central America (*40*).

The ability of some low pathogenicity avian influenza A viruses to bind to fucosylated receptors of the 3′SLe^X^ type has been considered a poultry-specific adaptation (*20**,**36**,**37**,**39**,**41*) although extensive studies on sufficient numbers of isolates across the complete range of HA genotypes are lacking. Except for low pathogenicity H7 viruses, which all bound efficiently to 6S-3′SLe^X^ irrespective of their host species (*42*), duck viruses were suggested to bind poorly to 3′SLe^X^−type receptors (*20**,**37**,**39**,**41**,**43*). However, adaptation to binding of 3′SLe^x^−type receptors has not been reported for high pathogenicity H5N1 viruses isolated from poultry.

A recent study showed poor binding of highly pathogenic H5 and H7 viruses to 3′SLe^X^ (*44*). In another study, binding of a few avian influenza A viruses to fucosylated receptors correlated with their binding to α2-6−linked sialosides (*41*). However, we found only weak binding of H5N8 virus HA to α2-6−specific transferrin (Figure 1, panel B) and no measurable binding to α2-6−linked sialosides in the glycan array or by biolayer interferometry (Figure 4). HAs of the Taiwanese/North American branch of clade 2.3.4.4 viruses did not bind α2-6−linked sialosides (*23*). Therefore, these results do not support the hypothesis that increased binding to fucosylated receptors enhances the propensity of avian influenza A viruses to evolve into binding α2-6−linked sialoside receptors.

Clade 2.3.4.4 viruses are generally considered to have evolved in and being spread by wild birds and ducks before introduction into poultry. Phylogenetic analyses based on HA sequences (Figure 4) have shown the evolvement into several branches/subclades often harboring multiple H5Nx virus genotypes. Substitutions K222Q and S227R were present in the earliest H5N5 virus isolates (A/duck/Guangdong/wy24/2008) and have been maintained in all branches except the Taiwanese/North American branch of clade 2.3.4.4 viruses, which obtained reverse substitution R227S. These findings strongly suggest that the capacity to bind to 3′SLe^X^−type receptors has arisen in wild birds.

The potential role of altered receptor specificity in extended host range and the contribution to the rapid worldwide spread of influenza viruses is still unknown. The presence of 3′SLe^x^−type receptors on intestinal epithelial cells varies between different avian species (Table 2) (*36*) and does not appear to be required for infection of these birds, but their presence in other tissues and species requires further investigation. Apart from determining host-range, altered receptor specificity might also influence other factors involved in spreading, such as virus titers, shedding, and pathogenesis in infected birds.

Outbreaks of clade 2.3.4.4 viruses in poultry might have contributed to enhanced spreading (*45**–**47*), However, adaptations in HA leading to evolvement of poultry-specific clades have not yet been detected in HA-based phylogenetic analyses. Reassortments of the 6 internal gene segments are continuously associated with the further evolution of H5Nx viruses, but the potential contribution of the internal gene constellation to (poultry-specific) spreading remains to be determined.

Of particular interest are the recent outbreaks of influenza caused by H5N6 viruses in poultry and ducks in Southeast Asia, which might resulted in nonavian infections, including 13, mostly lethal, cases in humans (*7*). Although enhanced avidity of these H5N6 viruses for human-type receptors (carrying α2-6−linked sialosides) has been reported (*22*), the amino acid combination Q222/R227, which all H5N6 viruses have in their HA, is unlikely to be responsible, and other amino acid substitutions, which have been shown to contribute to binding of α2-6−linked sialosides by highly pathogenic H5N1 viruses, have not been detected in H5N6 viruses (*48*,*49*). Two clades of H5N6 viruses have been identified by phylogenetic analysis (*50*), one harboring an NA with a truncated stem and the other harboring a full-length stem. Truncation of the stem has been considered a poultry-specific NA adaptation. However, both H5N6 virus clades appear to have acquired their N6 segment in independent events from H6N6 viruses (*50*), one of which already contained the stem deletion. Also, both clades have caused infections in wild birds, poultry, and humans, but evidence for species-specific adaptions in NA is lacking.

A longstanding paradigm in influenza A virus biology is the requirement for an optimal balance between HA binding and NA cleavage. HA binding displays a clear receptor fine specificity, but substrate fine specificity of NAs has not been extensively investigated. A recent report showed that all NA genotypes (only N4 was not tested) displayed relatively poor digestion of fucosylated receptors (*44*). Possibly because of tight binding of such receptors by clade 2.3.4.4 viruses, N1 of highly pathogenic H5 viruses might have lost an unknown advantage over other NA genotypes in creating an optimal HA/NA balance, which lead to the remarkable success of novel H5Nx virus reassortants within this clade.

Technical Appendix Additional information for highly pathogenic influenza A(H5Nx) viruses with altered H5 receptor-binding specificity.
