# Supplementary material for: Highly Pathogenic Influenza A(H5Nx) Viruses with Altered H5 Receptor-Binding Specificity
Source: Emerg Infect Dis. 2017 Feb;23(2):220–31. doi: 10.3201/eid2302.161072 (PMC5324792; doi:10.3201/eid2302.161072)
Supplement: Technical Appendix — Additional information for highly pathogenic influenza A(H5Nx) viruses with altered H5 receptor-binding specificity. [file 16-1072-Techapp-s1.pdf]

Article DOI: http://dx.doi.org/10.3201/eid2302.161072

Highly Pathogenic Influenza A(H5Nx) Viruses with Altered H5 Receptor-Binding Specificity

Technical Appendix

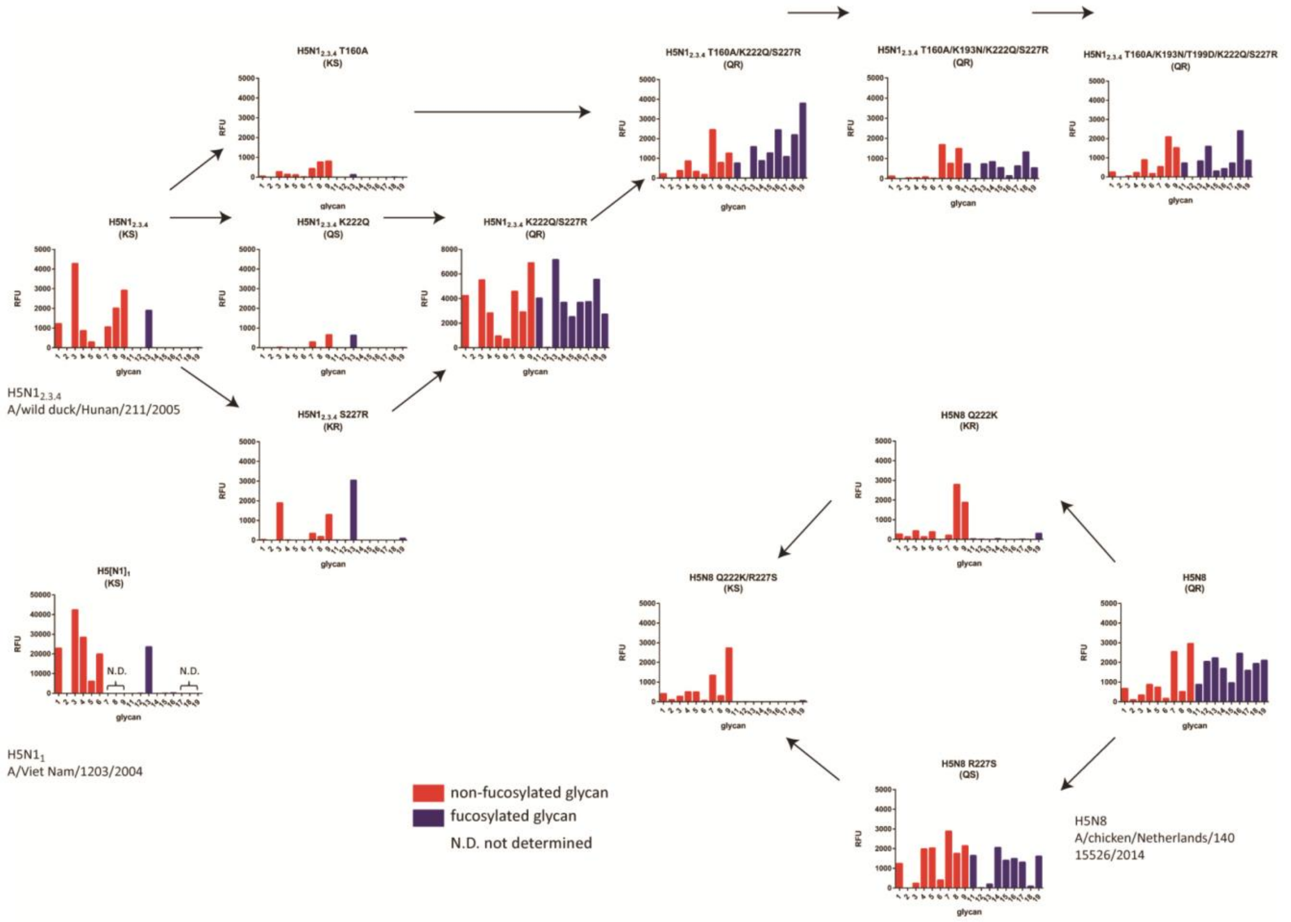

Technical Appendix Figure. Glycan array results for selected (non) fucosylated glycans for the complete set of wild-type and mutant H5 proteins of influenza viruses. Wild-type (H5N12.3.4, H5N8, and H5N11) and mutant H5 proteins (mutated residues are indicated) were applied to the glycan array precomplexed with StrepMAB-Classic (IBA GmbH, Göttingen, Germany) and fluorescent secondary antibodies. Letters in parentheses indicate amino acids at positions 222 and 227. Binding of hemagglutinin proteins is indicated in relative fluorescence units (RFU). Binding to sialylated glycans is shown for those present on the array in nonfucosylated (glycans 1–9; red bars) and fucosylated (glycans 11–19; blue bars) forms. Glycan numbers on the *x*-axes correspond to glycan structures shown in Figure 3 in the text. Arrows indicate the order by which the indicated substitutions were introduced in the recombinant proteins, which is based on phylogenetic analysis shown in Figure 5 of the text. Results for H5N1 virus were obtained from Wickramasinghe et al. (*1*). Glycans indicated by N.D. were not present in earlier arrays.

Reference

<jrn>1. Wickramasinghe IN, de Vries RP, Gröne A, de Haan CA, Verheije MH. Binding of avian coronavirus spike proteins to host factors reflects virus tropism and pathogenicity. J Virol. 2011;85:8903–12. PubMed http://dx.doi.org/10.1128/JVI.05112-11</jrn>
